# Immunostimulatory, anti-inflammatory, and immunomodulatory effects of Gum Arabic: regulation of cytokines and phagocytic function in rats

**DOI:** 10.3389/fimmu.2026.1812170

**Published:** 2026-04-13

**Authors:** Noof A. Alrabiah

**Affiliations:** Department of Biological Sciences, College of Science, King Faisal University, Al Ahsa, Saudi Arabia

**Keywords:** cytokines, Gum Arabic, innate immunity, neopterin, phagocytic index

## Abstract

**Introduction:**

The role of natural dietary fibers in supporting immune function and systemic physiological responses is increasingly recognized. This study evaluated the effect of long-term oral administration of gum arabic (GA) on the immune function of rats.

**Method:**

Seventy-six adult male Wistar rats were assigned to five groups: controls, GA treated (5% or 10% in drinking water for 6 weeks), and GA combined with LPS or dexamethasone, alongside corresponding LPS and dexamethasone controls.Phagocytic, histological, and immune markers were estimated and evaluated statistically by using one-way ANOVA followed by Duncan’s multiple range test.

**Results:**

GA treatment significantly increased serum complement-3 levels, lysozyme activity, immunoglobulin M (IgM), and nitric oxide (p < 0.05). GA also increased phagocytic index, neopterin, and intestinal mucosal eosinophil count, while spleen weight and mucosal mast cell count remained unchanged. GA mitigated the inhibitory effects of intramuscular dexamethasone on immune parameters and improved intravascular carbon clearance, and effectively attenuated the LPS induced increase in the pro-inflammatory cytokines (IL-1α, IL-4, IL-6, and TNF-α).

**Conclusion:**

These findings suggest that GA modulates selected immune parameters, enhances innate immune responses, and maintains systemic immune responsiveness under both normal and challenged conditions in rats.

## 1 Introduction

In recent decades, there has been a shift towards investigating natural products as potential candidates for alternative, complementary, and innovative therapeutic interventions. Some natural products have been reported to be well tolerated. However, they do not always cause fewer side effects, so their safety must be tested carefully (1). In addition, some may be more cost-effective and may improve patient adherence compared with conventional medications (2).

The exudate of Acacia species (Leguminosae) is a natural resin that contains arabin, a solidified, sticky fluid (3). Gum arabic (GA) was chosen because it is a natural food-grade biopolymer with a strong safety record. It is derived mainly from Acacia senegal and Acacia seyal, and acacia gum is affirmed as Generally Recognized as Safe (GRAS) for direct use in food under U.S. Food and Drug Administration regulation 21 CFR § 184.1330. Its established safety and wide use in food and pharmaceutical products support its selection for investigating possible immunomodulatory effects (4). GA is mainly derived from Acacia Senegal (90% yield) and Acacia seyal (10% yield) (5). It is synthesized through a natural process called gummosis, which occurs following injury to a tree’s bark. GA consists mainly of arabinogalactan, oligosaccharide, and glycoprotein (6). It is a complex of acidic heteropolysaccharides with a very high molecular weight. Because of its high solubility, GA forms a comparatively low-viscosity Newtonian solution, even at high concentrations (20-30% wt./wt). Metabolic studies show complete absorption of GA, with a caloric value of 4 calories/gram. Feeding 0.5 and 2.0g/day to weanling male Sprague-Dawley rats yielded caloric values of 131% and 110% of corn starch, respectively. GA administered at a dose of 50 g/day for 7 days prevented increases in triglyceride and cholesterol levels. Consequently, hyperlipemia was induced, which is believed to play a significant role in regulating hyperlipidemia in donkeys (7). GA has also helped reduce experimental toxicity by regulating the expression of oxidative stress genes to protect the liver (8).

As an edible substance, GA is widely used in food production, pharmaceutics, confectionery, cosmetics, pottery, textile, and beverages (9–11), working as a thickener, stabilizer, flavoring or coating agent, and emulsifier (12). In folk medicine, GA is used internally to treat intestinal mucosa inflammation and externally to cover inflamed surfaces (13). GA has also been reported to have antioxidant and nephroprotective properties (13–15). Since GA influences oxidative stress (16) and DNA damage (17), it can be used to treat gastrointestinal (18), cardiovascular (19), sickle cell anaemia (20), renal (15), and respiratory diseases. In addition to its direct biological effects, GA is recognized as a prebiotic fiber that is fermented by beneficial gut microbiota (21). By supporting intestinal health and improving the gut environment, GA may enhance nutrient utilization and help maintain immune balance. This gut-immune interaction may partly explain the reported immunomodulatory effects of GA. Moreover, GA protects against the effects of environmental and chemical hazards (22–24).

Several plant species contain compounds with immunomodulatory properties (25). These include the high-molecular-weight polysaccharide arabinogalactan (26), a distinct characteristic of GA. Widely popular as a resinous substance, GA has been used since ancient times as a chewing gum and as a beverage. Recently, GA has gained interest for its ability to influence functional immune parameters and is being evaluated in rat models. We hypothesized that GA may help regulate innate immunity in rats, under both normal conditions and during immune challenge. The novelty of this study is that it examined different immune-related parameters together, including serum biomarkers, phagocytic activity, cytokine responses, and intestinal findings, to provide a more comprehensive understanding of the immunological effects of GA.

## 2 Materials and methods

### 2.1 Extraction of gum

The *Acacia Senegal* (Active-Acacia, Sudanese gum arabic Company, Khartoum, Sudan) Gum arabic (GA) powder (4 g) was dissolved in 40 mL of 70% hydro-ethanol (*v*/*v*). Then it was covered with aluminum foil and extracted with a 40 kHz sonication power in an ultrasonic bath under the specified conditions (27). The extract was filtered and centrifuged at 15,000× *g* for 10 min. The solvent was discarded using a rotary vacuum evaporator at 40 °C. The extract was further freeze-dried and kept in a refrigerator until use.

The extraction yield (%) = (weight of extract after extraction (g)/weight of original sample (g)) x 100. Extraction yield was 74%, at the extraction time of 40 min, and the extraction temperature of 40°C.

### 2.2 Animals and treatments

The study was carried out using Adult Wistar male rats (6 weeks old and weighing 200-220g), which were kept at room temperature under a 12-hour light/dark cycle. Feed (according to the standard for mice and rats, ARASCO, Saudi Arabia) and tap water were provided *ad Libitum.*

A summary of the experimental design, including treatment groups, intervention conditions, and the number of animals allocated to each group, is shown in **Table 1**.

**Table 1:** Summary of the experimental design showing treatment groups, intervention conditions, and the number of animals allocated to each group in each experiment.

Experimental group	Number of animals	Description of treatment
A1	6	Untreated controls (phagocytic experiment)
A2	8	Untreated controls (serum used for immunological and histological parameters)
B1	6	Rats received 5% GA (for phagocytic experiment)
B2	8	Rats received 5% GA (serum used for immunological and histological parameters)
C1	6	Rats received 10% GA (for phagocytic experiment)
C2	8	Rats received 10% GA (serum used for immunological and histological parameters)
D1	8	Rats received 10% GA co-administered with LPS
D2	6	Rats injected with LPS
D3	6	Rats received saline (for control LPS experiment)
E1	8	Rats received 10% GA co-administered with dexamethasone (for phagocytic experiment)
E2	6	Rats received dexamethasone (for phagocytic experiment)

GA was given in drinking water to mimic regular oral consumption and to reduce stress associated with repeated gavage (28). The approximate daily intake was estimated from the measured water consumption of each group and the concentration of GA in the drinking water. This approach was considered more suitable for a long-term immunological study. The Institutional Animal Care and Use Committee at King Faisal University provided approval for all the procedures (approval number KFU-2025-ETHICS3660). In each case, blood samples were collected from the rats, and tissues were excised under anesthesia induced with 10 mg/kg xylazine and 50 mg/kg ketamine. For euthanasia, intraperitoneal sodium pentobarbital at a dose of 200 mg/kg body weight was used (29).

Seventy-six adult male Wistar rats were randomly divided into five groups. Group A (N = 14) served as untreated controls. Group B (N = 14) and C (N = 14) were treated with GA at doses of 5% and 10%, respectively, administered daily in drinking water for 6 weeks. The 5% and 10% GA doses were selected based on previously used experimental concentrations and to evaluate a possible dose-dependent response (30). In each group, 6 animals were used for the phagocytic assay, while the remaining 8 animals were sacrificed to collect blood and tissue samples for measurement of neopterin, other innate immune parameters, and for histological analysis. Group D1(N = 8) rats were given 10% GA co-administered with lipopolysaccharide (LPS) obtained from *E. coli* O111:B4 serotype (Sigma-Aldrich, Milan, Italy) freshly prepared in sterile pyrogen-free saline and injected intraperitoneally (IP) at a dose of 50 μg/kg (2 mL/kg body weight). Group D2 (N = 6) rats were treated with IP injection of LPS at a dose of 50 μg/kg, and Group D3 (N = 6) rats were treated with IP saline injections (2 mL/kg body weight), and were used to keep a check on the serum cytokine levels. Group E1 (N = 8) rats were given 10% GA co-administered with dexamethasone sodium phosphate (Decadron, Merck Sharp and Dohme, Herts, UK) at 1mg/kg body weight, injected intramuscularly. Group E2 (N = 6) rats received dexamethasone alone. Phagocytic experiments were then performed on these animals.

The tissues were kept in liquid nitrogen. Venipuncture, centrifuged at 1500g, was used to obtain jugular blood from all animals in different experiments, and serum was kept at -30˚C until analysis.

### 2.3 Measurement of lysozyme, immunoglobulin M, nitric oxide, and complement 3

Turbidimetric assays were used to determine lysozyme activity (36). The concentrations of Serum immunoglobulin M (IgM), nitric oxide (NO), and complement 3 (C3) were measured using ELISA kits from MyBioSource in San Diego, USA, in accordance with the manufacturer’s instructions.

### 2.4 Measurement of mononuclear phagocytic function

To determine rat MPF, the intravascular clearance of carbon colloid was measured according to the guidelines of Al-Afaleq and Homeida (31). Animals were anesthetized as previously described. After excising the trachea, a catheter (Angiocath, 16 gauge; Deseret Medical, Sandy, UT, USA) was inserted into it. Catheters (Angiocath, 24 gauge) were also inserted into the right and left femoral veins. Colloid carbon was administered intravenously at a dose of 0.08 mg/kg body weight (Gunther, Wagner, Hanover) into the right femoral vein. The left femoral vein was used to obtain the serial blood samples. To haemolyze the blood, 4 mL of 0.1% sodium carbonate was used, after which the samples were centrifuged at 500 g for 5 minutes. To estimate the relative amount of carbon remaining in the supernatant, the samples obtained before carbon injection were assigned a value of zero. A graph was plotted against time in minutes, with carbon concentrations represented as a percentage of the injected dose on a semi-logarithmic scale, which allowed the intravascular half-life (t _1/2_) to be computed in minutes. Absorbance was also plotted against time, and phagocytic index was determined (32) using the formula: Phagocytic index = Rct/Rcc, where Rct and Rcc signify regression coefficient (treatment) and regression coefficient (control), respectively.

### 2.5 Histological studies

After the experiment ended, mucosal mast cells and eosinophils were counted. To measure mucosal mast cells, tissues of the small intestines were fixed using Carnoy’s fixative, and Alcian blue, pH 0.3, and Safranin-O, pH 0.1, were used to stain paraffin-embedded sections (33). Intra-epithelial mast cells were counted in 50 villus-crypt units (VCU) and denoted as mast cell numbers per 10 VCU. Tissue samples for eosinophil detection were fixed in acetone, and hematoxylin was used to stain paraffin-embedded sections, followed by 1% water-soluble Biebrich Scarlet (Sigma) for 5 minutes (34). Eosinophils in the small intestine were counted in 50 VCU and denoted as the eosinophil number per 10 VCU.

### 2.6 Cytokine measurements

ELISA kits were used to measure serum cytokines and immune markers following the manufacturer’s instructions. ELISA kits specific for rats (TFBCO, Tokyo, Japan) were used to measure the concentrations of serum cytokines IL-1α, IL-4, IL-6, and TNF-α at absorbance of 450 nm, using a microplate reader (Bio-Tek Instruments, Inc., Winooski, USA). The assays had sensitivities of 4.1, 0.20, 12, and 11.2 pg/ml, respectively. The inter-assay coefficient of variation was lower than 5%. All samples were analyzed under the same conditions, standard curves were prepared for each assay, and samples were tested in duplicate to improve reliability.

### 2.7 Statistical analysis

Statistical analysis was performed using SPSS version 25.0 (IBM Corp., Armonk, NY, USA). Results are expressed as mean ± SD. Differences among experimental groups were analyzed by one-way ANOVA, followed by Duncan’s multiple-range test for *post-hoc* comparison of group means when significant differences were observed (35). A p-value of < 0.05 was considered statistically significant.

## 3 Results

**Figure 1:**
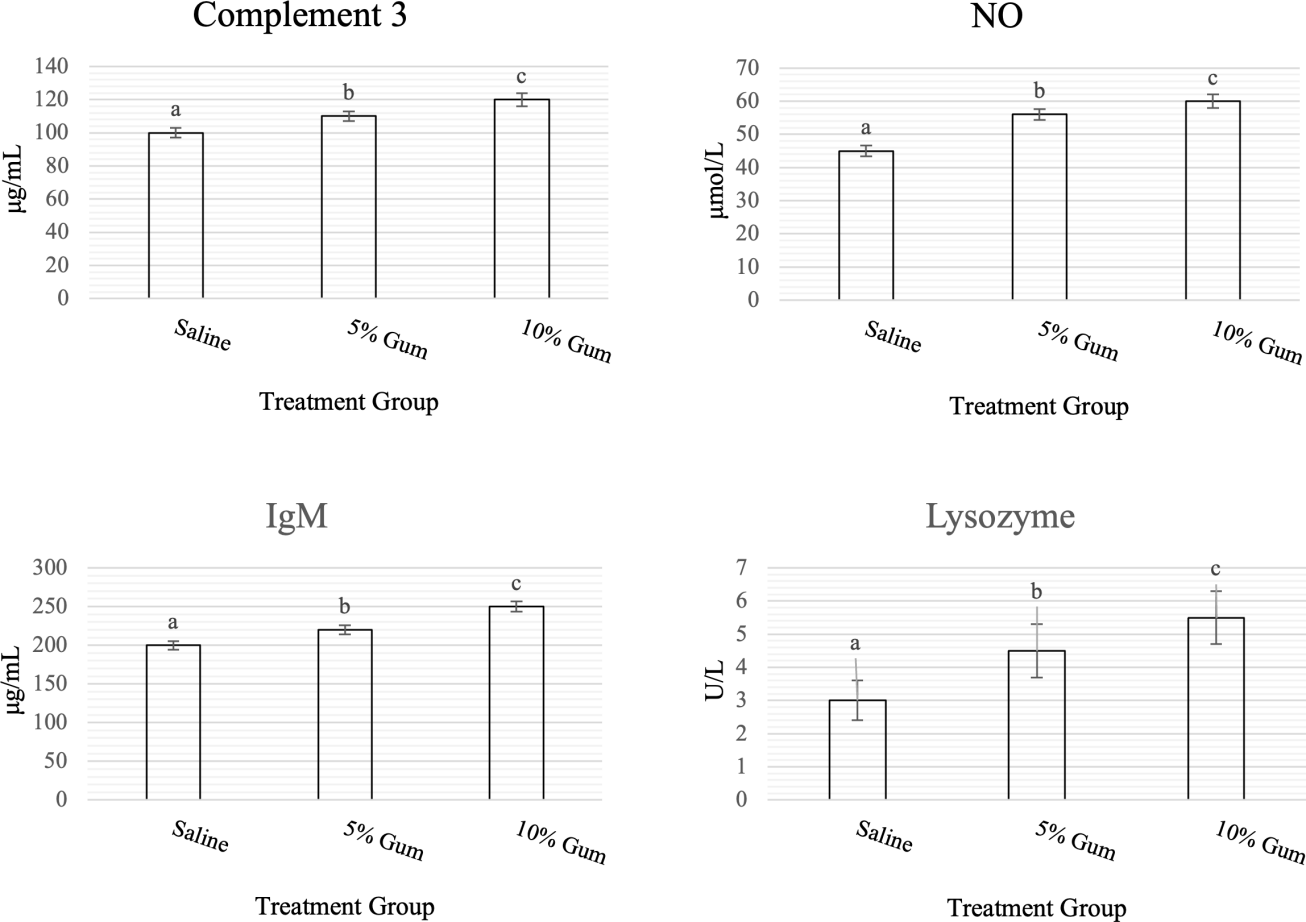
Effect of orally administered GA on innate immune parameters of rats for 6 weeks. Data are expressed as mean (N = 8) ± SD. Columns bearing different letters are significantly different at p<0.05.

**Figure 1** shows the impact of administering GA on the innate immune parameters of rats. The effect of Gum on serum lysozyme, IgM, NO, and C3 in rats was significant (p < 0.05).

**Table 2** shows the impact of administering saline, Gum, and dexamethasone on the phagocytic index, spleen weight, count of mucosal mast cells, and eosinophils and plasma concentrations of neopterin in rats.

**Table 2:** Effects of saline, gum, and dexamethasone treatments on various immune parameters in rats.

Parameter	Saline	Gum	Dexamethasone	Dexamethasone + gum
Phagocytic Index	1.00^a*^	1.40^b^	0.45^c^	0.81^a^
Neopterin (nmol/L)	4.12 ± 0.61^a^	6.30 ± 0.71^b^	2.60 ± 0.38^c^	4.60 ± 0.44^a^
Spleen weight (g)	0.30 ± 0.10^a^	0.29 ± 0.10^b^	0.17 ± 0.04^b^	0.26 ± 0.10^a^
MMC (count/10VCU)	138 ± 17^a^	141 ± 16^a^	82 ± 8^b^	123 ± 17^a^
Eosinophil (count/10VCU)	36 ± 3^a^	56 ± 4^b^	21 ± 2^c^	31 ± 3^a^

*Values within the same row that have different superscript letters (a, b, c) are significantly different at (*p<0.05*). MMC, mucosal mast cells; VCU, villus-crypt units.

The plasma concentration of neopterin increased significantly by 53% (from 4.12 ± 0.61 to 6.30 ± 0.71 nmol/L; p<0.05) following Gum administration (p<0.05) in rats compared to control rats treated with saline. After dexamethasone administration, neopterin concentration decreased significantly (p<0.05) to 2.60 nmol/L.

However, when Gum and dexamethasone were administered together, neopterin levels increased by 77% (from 2.60 ± 0.38 to 4.60 ± 0.44 nmol/L; p<0.001) compared with dexamethasone-treated rats (p<0.001). Their levels were identical to those of saline-treated control rats.

Spleen weight was similar in rats treated with saline, Gum, and dexamethasone-gum groups (0.30g, 0.29g and 0.26g, respectively). However, it was significantly higher (p<0.05) than in rats treated with dexamethasone alone (0.17g). Rats treated with GA showed a significantly higher mucosal eosinophil count in intestinal tissue sections compared with controls, increasing by 56% (from 36 ± 3 to 56 ± 4 count/10VCU; p<0.05), as assessed histologically per 10 *villus-crypt units*. dexamethasone reduced eosinophil counts to 21 ± 2 (count/10VCU). While co-treated with gum partially restored eosinophil counts to 31 ± 3 (count/10VCU). Mast cells remained unchanged in the GA-treated group compared with controls. However, in rats treated with dexamethasone and Gum, mast cell counts increased significantly by 50% (from 82 ± 8 to 123 ± 17; p<0.01) compared with rats treated with dexamethasone alone.

The phagocytic index of the dexamethasone group was 0.45, which was significantly (p<0.05) lower than that of control animals. In normal rats, Gum produced a significantly (p<0.05) higher index (1.40), while it was even higher (p<0.001) in those treated with dexamethasone-gum (0.81).

**Figure 2:**
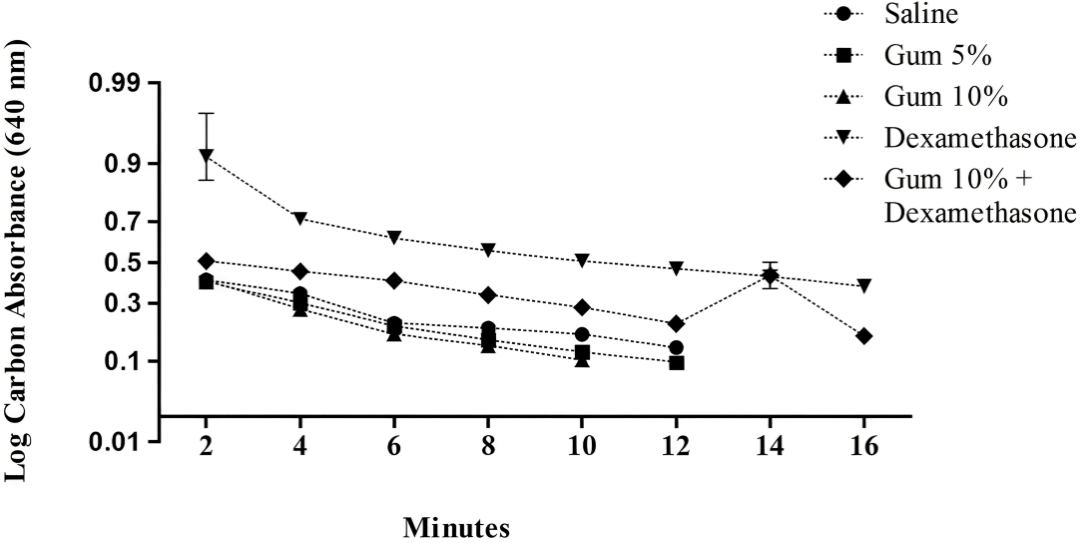
Effect of saline, gum, and dexamethasone treatments on plasma carbon clearance in rats.

**Figure 2** illustrates how Gum, dexamethasone, and saline influence plasma carbon clearance. Compared to controls, Gum increased intravascular carbon clearance (p<0.05) and decreased the vascular half-life. Conversely, dexamethasone caused a significant (p=0.05) reduction in carbon clearance compared to controls.

The values of clearance rates were as follows: 8.4 ± 0.3 saline-treated, 6.5 ± 0.30 for 5% Gum-treated, 5.2 ± 0.40 for 10% Gum-treated, 12.6 ± 1.2 for dexamethasone, and 9.0 ± 61.2 minutes for dexamethasone-gum-treated rats.

**Figure 3:**
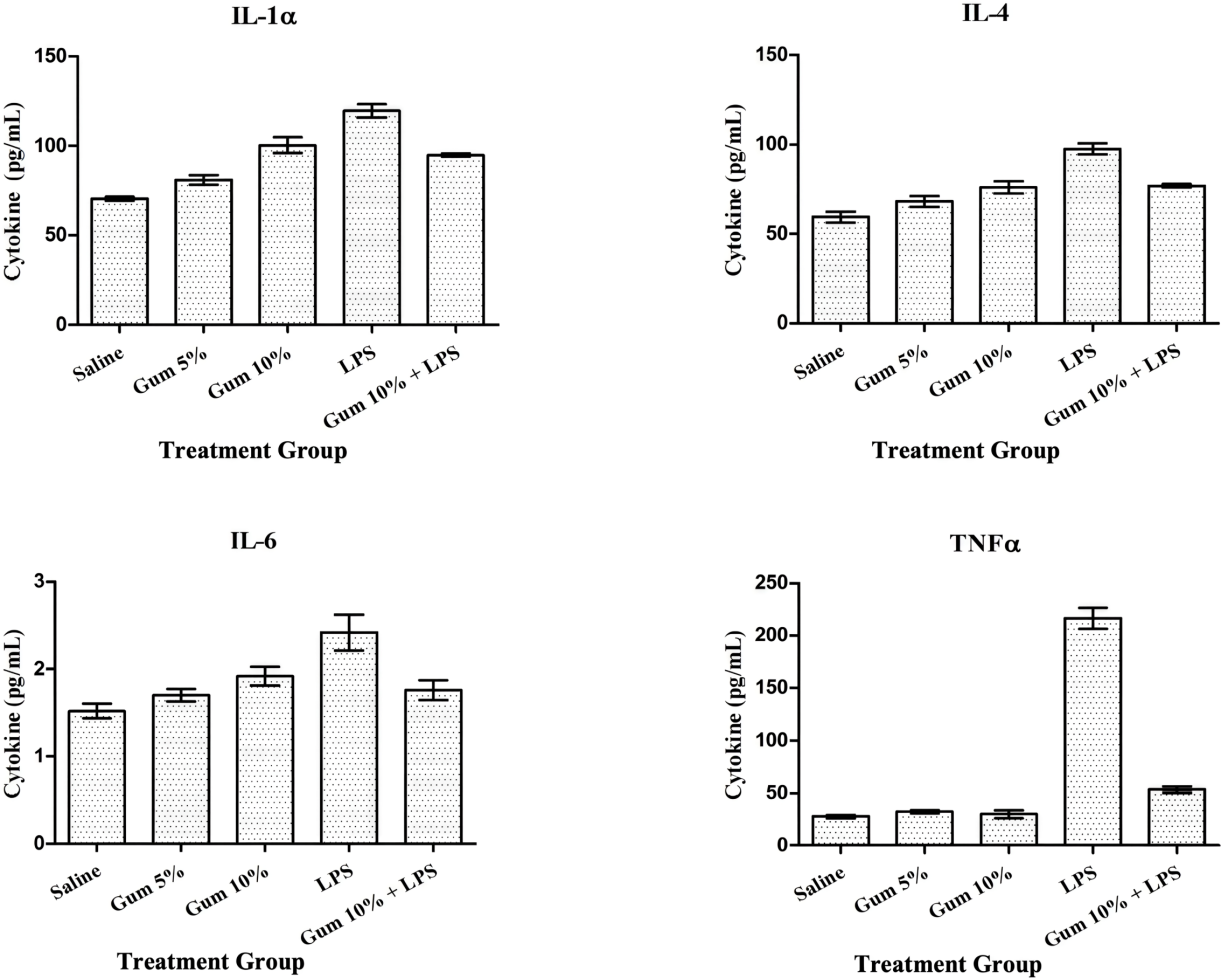
TNF-α, IL-1α, IL-4, and IL-6 concentrations in the serum of rats treated with saline, 5% and 10% gum, lipopolysaccharide (LPS), and LPS-Gum (10%), respectively.

**Figure 3** demonstrates the impact of saline, 5% and 10% Gum, LPS, and LPS co-administered with Gum on cytokine concentration.

In comparison to controls, Gum at a 10% dose significantly increased (p<0.05) plasma concentration of IL-1α by 42% (70.3 pg/ml in saline treated vs 99.8 pg/ml in gum treated), IL-4 (55.0 pg/ml in saline treated vs 73.1 pg/ml in gum treated) and IL-6 (1.56 pg/ml in saline treated and 2.07 pg/ml in gum treated) by 33%, with no impact on TNF. LPS treatment cause a significant increase (p<0.01) in IL-1 (70.3 pg/ml in saline treated vs 138.49 pg/ml in LPS treated), IL-4 (55 pg/ml in saline treated vs 91.3 pg/ml in LPS treated), IL-6 (1.56 pg/ml in saline treated vs 2.58 pg/ml in LPS treated), and TNF-α (30 pg/ml in saline treated and 210 pg/ml in LPS treated) by 97%, 66%,65% and 600%, respectively.

In addition, compared with rats treated with LPS alone, Gum at a 10% dose administered with LPS results in a significant (p<0.05) decline in IL-1, IL-4, IL-6, and TNF-α by 28%, 40%, 38%, and 78%, respectively.

## 4 Discussion

Administering GA to rats caused a significant increase in serum levels of innate immune biomarkers, such as complement 3, phagocytic activity, NO, lysozyme, IgM, and neopterin, compared to saline-treated control rats. These immune markers help assess the non-specific immune response to natural products (37). There is a close link between cellular immune system activation (38) and neopterin biosynthesis, which is also associated with immune responses, such as viral infections (39, 40). It was observed that patients with viral infections had higher neopterin concentrations, indicating that the increased levels may reflect an immune response activated against virus-infected cells (41). Neopterin is released into the cell culture medium following antigenic stimulation of human peripheral blood reticuloendothelial/mononuclear cells. These human macrophages produce neopterin *in vitro* upon interferon stimulation (42). Depending on its ease of measurement, neopterin is now regarded as a pro-inflammatory immune status (38, 41). The administration suppressed the release of neopterin. However, when GA was administered along with dexamethasone, it entirely prevented dexamethasone inhibition following neopterin release.

According to the U.S. Food and Drug Administration, GA is considered one of the safest dietary fibers (4). Therefore, its potential effects on the local intestinal immune system were investigated. In comparison to controls, mucosal eosinophil count increased significantly in rats treated with GA, while mast cells remained the same. Furthermore, GA blocked the immunosuppressive effects on intestinal mucosal cell counts caused by dexamethasone, which implied the role of GA in intestinal immunity. In various experimental models, eosinophilia and mast cells have been shown to play an essential role in host protection (43). A strong association has been reported between the expulsion of *Strongyloides* worms from mice and the proliferation and activation of mast cells and eosinophils (44–47).

Similar spleen weights were observed in rats treated with saline, Gum, and dexamethasone-Gum, and these were significantly higher (p<0.05) than in rats treated with dexamethasone. The current study notes that spleen size decreased in the dexamethasone group, aligning with reports by Davison et al. (48), which support the idea of reduced spleen weight in immunosuppressed animals (49).

According to the data obtained, dexamethasone-treated rats exhibited a significant suppression of reticuloendothelial/mononuclear phagocyte function. Following Gum administration (alone or along with dexamethasone), the phagocytosis efficiency to eliminate foreign particles like carbon colloid may not be due to the stimulation of the reticuloendothelial system function. This was evident from the decrease in intravascular carbon clearance and vascular half-life compared to controls. Phagocytosis is a complex process that involves opsonization, followed by receptor-mediated recognition and attachment to the macrophage surface (50), endocytosis, and ultimately digestion (51). The result section presents the phagocytic indices of the various groups. The mononuclear phagocytic function of the dexamethasone group decreased, as implied by the value of 0.45 shown by this group. In normal rats, Gum produced a significantly greater index (1.40), which was even higher than the rats treated with dexamethasone-gum (0.81).

The study aimed to determine the cytokine pattern in rats undergoing GA treatment, including Th1 cytokines (IL-6 and TNF-α), Th2 cytokines (IL-4), and the pro-inflammatory cytokine IL-1 produced by macrophages. Administering GA to rats induced the production of IL-1, IL-4, and IL-6. Similarly, Gum treatment led to cytokine synthesis, which are vital regulators of immune response (52–54). GA treatment interestingly induces the release of the pro-inflammatory cytokines IL-1 and IL-6, which in turn increase the proliferation of T-lymphocytes, antibody production, synthesis of adhesion molecules, and also of acute-phase proteins (55, 56). These cytokines exhibit certain overlapping functions, e.g., endogenous pyrogenic effect, activation of macrophages, and co-stimulation of T-lymphocytes (57). Moreover, GA treatment simultaneously induced IL-4 release, which can suppress pro-inflammatory cytokine activity, inhibit T-lymphocyte proliferation, and participate in immune responses to different antigens (58). In addition, it helps resolve inflammation through a series of regulatory mechanisms (59).

LPS further increased cytokine synthesis. It has also been reported to stimulate macrophages to release IL-1 and TNF-α, thereby regulating the host’s biological response to endotoxin exposure (60). E. coli infection causes the release of IL-6 in baboons (61), which potentially induces acute phase proteins in the liver (62).

Some previous studies suggest that GA may act by affecting NF-κB, the gut microbiota, and butyrate production (63–65). However, these mechanisms were not directly investigated in this study. Therefore, their contribution to our findings cannot be confirmed, and further studies are needed to clarify these potential pathways. Cytokine levels decreased following the co-administration of Gum and LPS compared with the group that received LPS alone. This suggests that GA may help protect against sepsis complications.

This study has several limitations. One limitation is that eosinophil-related findings were based solely on histological analysis of tissue sections, and no serum or peripheral blood eosinophil markers were measured to confirm systemic effects. In addition, the serum biomarker results suggest that GA may affect innate immunity. The intestinal findings also point to a possible role of the gut in this effect. Because GA is a prebiotic, it may work partly by supporting beneficial gut bacteria. However, since the gut microbiota was not directly analyzed, this explanation remains possible rather than proven.

## 5 Conclusion

In conclusion, this study provides new evidence that GA can influence immune-related parameters in rats. A main strength of the work is that it combines serum innate immune biomarkers with intestinal findings, giving a broader view of its possible effects at both the systemic and gut levels. These results improve our understanding of the immunomodulatory activity of GA in this experimental model. Further studies are needed to confirm the underlying mechanisms, especially the possible role of the gut microbiota.

## Data Availability

The original contributions presented in the study are included in the article/supplementary material. Further inquiries can be directed to the corresponding author.
